# MicroRNA gga-miR-200a-3p modulates immune response via MAPK signaling pathway in chicken afflicted with necrotic enteritis

**DOI:** 10.1186/s13567-020-0736-x

**Published:** 2020-02-03

**Authors:** Thu Thao Pham, Jihye Ban, Yeojin Hong, Jiae Lee, Thi Hao Vu, Anh Duc Truong, Hyun S. Lillehoj, Yeong Ho Hong

**Affiliations:** 1 Department of Animal Science and Technology, Chung-Ang University, Anseong, 17546 Republic of Korea; 2 Department of Biochemistry and Immunology, National Institute of Veterinary Research, 86 Truong Chinh, Dong Da, Hanoi, 100000 Viet Nam; 3 Animal Biosciences and Biotechnology Laboratory, Agricultural Research Services, United States Department of Agriculture, Beltsville, MD 20705 USA; 4 Key Laboratory of Animal Cell Biotechnology, National Institute of Animal Science, 9 Tan Phong, Thuy Phuong, Bac Tu Liem, Hanoi, 100000 Viet Nam

## Abstract

MicroRNAs (miRNAs) are small non-coding RNAs that contribute to host immune response as post-transcriptional regulation. The current study investigated the biological role of the chicken (*Gallus gallus*) microRNA-200a-3p (gga-miR-200a-3p), using 2 necrotic enteritis (NE) afflicted genetically disparate chicken lines, 6.3 and 7.2, as well as the mechanisms underlying the fundamental signaling pathways in chicken. The expression of gga-miR-200a-3p in the intestinal mucosal layer of NE-induced chickens, was found to be upregulated during NE infection in the disease-susceptible chicken line 7.2. To validate the target genes, we performed an overexpression analysis of gga-miR-200a-3p using chemically synthesized oligonucleotides identical to gga-miR-200a-3p, reporter gene analysis including luciferase reporter assay, and a dual fluorescence reporter assay in cultured HD11 chicken macrophage cell lines. Gga-miR-200a-3p was observed to be a direct transcriptional repressor of *ZAK*, *MAP2K4*, and *TGFβ2* that are involved in mitogen-activated protein kinase (MAPK) pathway by targeting the 3′-UTR of their transcripts. Besides, gga-miR-200a-3p may indirectly affect the expression of protein kinases including *p38* and *ERK1/2* at both transcriptional and translational levels, suggesting that this miRNA may function as an important regulator of the MAPK signaling pathway. Proinflammatory cytokines consisting of *IL*-*1β*, *IFN*-*γ*, *IL*-*12p40*, *IL*-*17A*, and *LITAF* belonging to Th1 and Th17-type cytokines, were upregulated upon gga-miR-200a-3p overexpression. These findings have enhanced our knowledge of the immune function of gga-miR-200a-3p mediating the chicken immune response via regulation of the MAPK signaling pathway and indicate that this miRNA may serve as an important biomarker of diseases in domestic animals.

## Introduction

Necrotic enteritis (NE), a disease which occurs primarily in avian species, is caused by high levels of *Clostridium perfringens* (*C.P*) [1]. *C.P* is a gram-positive, spore-forming anaerobe typically found in low abundance (< 10^4^ cfu) in the gastrointestinal tract (GIT) of most bird species [2]. However, excessive *C.P* counts, particularly in the small intestine, lead to the onset of NE [3, 4]. *C.P* produce toxins and the intestinal mucosa may be covered with a fibrino-necrotic layer [5]. Such gut epithelial damage is frequently associated with coccidiosis caused by the coccidian genus, *Eimeria* [6]. NE outbreaks commonly occur in 17–18 days old broiler chickens [7]. Affected birds show symptoms such as huddling, ruffled feathers, inappetence, lowered growth rates, feed conversion efficacy, and diarrhea, which leads to high mortality rates [5, 8, 9]. At first, the use of antimicrobials such as antibiotic growth promoters (AGPs) and other therapeutic agents effectively reduced NE, and they were used worldwide. However, in response to emerging concerns regarding antimicrobial resistance, the use of antimicrobials in poultry production has been banned from 2006 in the EU and from 2012 in Korea [10]. Thus, effective new methods capable of controlling NE, which causes serious economic loss and affects animal welfare, are needed. In addition, research investigating immunological and pathological avian host response to *C.P* and *Eimeria maxima* (*E.M*) has been limited.

Several functional studies on miR-200a have been conducted in vertebrates. In human and mice, it was reported that *MALAT1* affected proliferation, migration, invasion, and apoptosis during the progression of hypoxic hepatocellular carcinoma by sponging miR-200a [11]. Moreover, miR-200a mediated the proliferation of hepatic stellate cells and development of fibrosis by targeting the 3′-UTR of *SIRT1* via the SIRT1/Notch signal pathway [12]. It was also involved in protecting thymosin β-4 in cardiac microvascular endothelial cells following hypoxia/reoxygenation injury via the *NRF2* antioxidant pathway [13]. Moreover, expression of miR-200a was downregulated in fibrostenosing Crohn’s disease [14], HBV-induced hepatocellular carcinoma [15] and human glioma [16], thereby highlighting its function as a suppressor of many diseases. In chicken, gga-miR-200a regulated cell differentiation and proliferation of breast muscle by target 3′-UTR of *GRB2* [17]. Additionally, gga-miR-200-3p was expressed in high abundance between 14 weeks and 22 weeks, and it also targeted *TGFB3* related to TGF-beta signaling pathway and MAPK signaling pathway in abdominal adipose tissue during postnasal late development [18]. In response against Reticuloendotheliosis Virus, gga-miR-200a-3p was negatively correlated with *CTLA4*, a negative regulator of T cell activation in spleen [19]. Interestingly, gga-miR-200a-3p was found to be significantly up-regulated in the susceptible line 7.2 compared to the resistant line 6.3 in response to Necrotic Enteritis in our previous study [20]. However, the regulatory mechanism of gga-miR-200a-3p in the immune response has remained uninvestigated.

The current study analyzed the biological role of gga-miR-200a-3p as a regulator of chicken immune response and investigated its target genes, with a specific focus on those involved in the MAPK signaling pathway. We focused on gaining a better understanding of miR-200a expression following NE-induction in 2 highly inbred chicken lines and validated the target genes of gga-miR-200a-3p using a chicken macrophage cell line, HD11.

## Materials and methods

### Necrotic enteritis disease model animals

Two White Leghorn chicken lines, line 6.3 and line 7.2, which have been highly inbred and maintained since 1931, show resistance or susceptibility to avian leucosis virus (ALV) and Marek’s disease virus (MDV), respectively [21]. To induce NE, the chickens were challenged with *E.M* strain 41A (1.0 × 10^4^ oocysts/birds) by oral gavage at day 14 after hatching, followed by challenge with *C.P* strain Del (1.0 × 10^9^ cfu/bird) by oral gavage for the next 2 days, (day 4 following *E.M* infection). The infection experiment was extended for 6 days. Intestinal mucosal layers (IMLs) were collected from 5 chickens per group following NE induction. The IMLs samples were provided by the Animal Biosciences and Biotechnology Laboratory (Beltsville, MD, USA) of the United States Department of Agriculture (USDA)-Agricultural Research Service. All animal protocols were approved by the Institutional Animal Care and Use Committees of the Beltsville Agricultural Research Center (Protocol #09-019). The IMLs were carefully homogenized after freezing with liquid nitrogen, and total RNA was extracted using TRIzol (Invitrogen, Carlsbad, CA, USA).

### Target gene prediction of gga-miR-200a-3p

Prediction of the target genes of gga-miR-200a-3p was carried out via miRDB v6.0 [22], which contains chicken miRNA as well as mRNA data, and provides a custom prediction mode based on mature miRNAs sequences. Genes with a target score of more than 80 were further functionally analyzed using the DAVID Bioinformatic Resources [23, 24] and KEGG PATHWAY Database [25], leading to the mapping of genes involved in immune-related pathways, such as the MAPK signaling pathway, TGF-beta pathway and/or toll-like receptor signaling pathway. Next, potential targets were predicted based on their meeting these criteria and on the basis of the presence of gga-miR-200a-3p binding sites in their 3′-UTR [26].

### Cell culture, mimic miRNA and LPS stimulation

The HD11 chicken macrophage cell line [27] was cultured in RPMI-1640 medium, supplemented with l-glutamine (Gibco, Grand Island, NY, USA), 10% heat-inactivated fetal bovine serum (Gibco) and 1% penicillin–streptomycin (Gibco). The cells were incubated under humidified conditions in a 5% CO_2_ atmosphere at 41 °C.

Based on the precursor sequence of gga-miR-200a-3p (MI0001249) obtained from miRbase catalogs [28], an oligonucleotide gga-miR-200a-3p mimic was chemically synthesized by Bioneer (Daejeon, Republic of Korea) for overexpression of the miRNA in cells. The experimental group consisted of 4 treatments as follows: (1) control; (2) exposure to lipopolysaccharide (LPS) of *Salmonella enteritis* (*S.E*); (3) mimic miR-200a overexpression; and (4) mimic miR-200a overexpression upon exposure to LPS.Each group consisted of 3 independent replicates. To overexpress the mimic miRNA, synthetic mimic gga-miR-200a-3p oligonucleotides were transiently transfected into the HD11 chicken macrophage cell line seeded in a 12-well plate at a final concentration of 50 nM using Lipofectamine 3000 (Invitrogen, Carlsbad, CA, USA) diluted in Opti-MEM medium (Gibco), according to the manufacturer’s instructions. Following 20 h of transient transfection, either LPS (1.0 µg/mL) (Sigma-Aldrich, St. Louis, MO, USA) or normal medium were used to stimulate the treated cells for the 4 h. The control group was treated with similar amounts of Lipofectamine 3000 (Invitrogen) and Opti-MEM (Gibco) without the oligonucleotides. Samples were collected from each group for further experimentation.

### Construction of gga-miR-200a-3p or candidate target gene 3′-UTR constructs

The oligonucleotide sequence of mature gga-miR-200a-3p was cloned into a pDsRed2-N1 plasmid, containing a cytomegalovirus (CMV) promoter carrying the red fluorescence protein (RFP) (Clontech, Palo Alto, CA, USA), using NotI digestion (5′ and 3′) (Additional file 1). This construct, named miR200a/DsRed simultaneously expressed a small fragment of miRNA molecule and RFP for visualizing the RNA molecule. Regions of the 3′-UTR flanking the predicted miR-200a binding sites in the candidate target genes were amplified using pooled cDNA of ADOL chicken lines as templates; the specific forward and reverse primers used have been provided (Additional file 1). For the dual fluorescence assay, the partial 3′-UTR of *TGFβ2* was ligated into a pcDNA3 plasmid, containing enhanced green fluorescence protein (eGFP) using NotI/XbaI restriction enzymes. The resulting construct was called TGFβ2/EGFP. For the luciferase assay, the target genes PCR products were digested via SacI/HindIII and cloned into a pMIR-REPORT Luciferase vector (Ambion, Austin, TX, USA), resulting in “Gene”/Luc recombinant constructs independently containing *TGFβ2*, *ZAK* and *MAP2K4.* The ligated constructs were transformed into top 10 competent *Escherichia coli* (*E. coli*) (Invitrogen) and recombinant plasmids were confirmed by sequencing (Genotech, Daejeon, Republic of Korea).

### Quantitative real-time PCR (qRT-PCR) for mRNA and miRNA

Total RNA from chicken lines 6.3 and 7.2, and HD11 cells was isolated using Trizol reagent (Invitrogen) in accordance with the manufacturer’s instructions. cDNAs from mRNA were synthesized using a RevertAid First Strand cDNA Synthesis kit (Thermo Scientific, Waltham, MA, USA). In brief, the reaction mixture (20 μL) contained RNA (2 μg), 5× Reaction Buffer (4 μL), 10 mM dNTP Mix (2 μL), Oligo (dT)_18_ primers (1 μL), RiboLock RNase Inhibitor (1 U), and RevertAid M-MuLV RT (10 U). The reaction was subjected to the following conditions: 60 min at 42 °C, followed by 70 °C for 5 min. For miRNA cDNA synthesis, a Ncode™ miRNA First-strand cDNA synthesis Kit (Invitrogen) was used as per the manufacturer’s protocol. Diluted amounts of cDNA were used as templates to perform quantitative RT-PCR (qRT-PCR) as per manufacturer’s instructions. Specific forward primers for miRNAs were designed and a universal primer was used as the reverse primer. Primers for mRNAs were designed using Primer-BLAST [29] (Additional file 1). qRT-PCR was performed using 2× Power SYBR Green Master Mix (Roche Life Science, Mannheim, Germany) and the LightCycler^®^ 96 System (Roche Life Science) in accordance with the manufacturer’s instructions. Threshold cycle (Ct) values were normalized to those of GAPDH (mRNA) or U1A small nuclear RNA (miRNA) by the 2^− ΔΔCt^ method [30].

### Western blotting

The HD11 chicken macrophage cells transfected with gga-miR-200a-3p mimic or gga-miR-200a-3p mimic in the background of endotoxins LPS stimulation were analyzed for protein expression. Protein samples, the concentrations of which were measured by a BCA assay, were electrophoresed on SDS-PAGE gels containing 10% polyacrylamide and transferred onto polyvinylidene difluoride (PVDF) membranes (GE Healthcare, Rydalmere, Australia). The PVDF membranes were blocked with 5% non-fat milk containing 0.05% Tween 20 in PBS (pH 7.4) (PBST) for 1 h. Primary antibodies including rabbit anti-chicken phosphor-p44/42 MAPK (Cell Signaling Technology, ERK1/2; Thr202/Tyr204, #4370, specific), rabbit anti-chicken phospho-p38 MAPK (Cell Signaling Technology, Thr180/Tyr182, #4631, specific), and mouse anti-chicken GAPDH antibodies (Thermo Fisher Scientific, AM4300, specific) were prepared based on the specific dilution ratio of each primary antibody in PBST containing 2% non-fat milk for overnight incubation with the PVDF membranes at 4 °C. After washing with PBST, membranes were treated with horseradish peroxidase (HRP)-linked anti-rabbit or anti-mouse (Thermo Fisher Scientific) secondary antibodies (based on the primary antibodies) diluted in PBST containing 2% non-fat milk and incubated for 2 h at room temperature. Band signals were detected using Western Lightning ECL Plus substrate (Thermo Fisher Scientific) and were exposed on Hyperfilm (GE Healthcare).

### Dual fluorescence assay

The HD11 macrophage cells were seeded at 1.0 × 10^6^ cells/well and cultured in 12-well plates (Corning, NY, USA). When cells reached 80% confluency, the cells were transfected with miR200a/DsRed or TGFβ2/EGFP, or co-transfected with both plasmids using Lipofectamine 3000 (Invitrogen) as per the manufacturer’s instructions. Three hours after transfection, 50 µM of β-mercaptoethanol was added to each well. Following 48 h of transfection, eGFP or DsRed were visualized using an EVOS^®^ FL Color Imaging System (Life Technologies). The settings of the fluorescence microscope were as follows: 470 nm excitation filter and 525 nm emission filter for green fluorescent protein; and 530 nm excitation filter and 593 nm emission filter for red fluorescent protein.

### Luciferase reporter assay

The HD11 macrophage cells were seeded at 1.0 × 10^6^ cells/well and cultured in 12-well plates (Corning). “Gene”/Luc were co-transfected along with miR200a/DsRed into the cells using Lipofectamine 3000 (Invitrogen) as per the manufacturer’s instructions; the pMIR-REPORT β-gal control plasmid (Ambion) was also transfected to normalize transfection efficiency. The cells were collected after 24 h and lysed by 1× luciferase cell culture lysis reagent (Promega). After centrifugation at 20 000 × *g* for 1 min, the supernatant was used for the measurement of the luciferase and β-galactosidase activity in 96-well plates (Corning) using Luciferase Assay Systems (Promega, Madison, WI, USA) and β-galactose solution (o-nitrophenyl-β-d-galactopyranoside or OPNG), respectively, as per the manufacturer’s instructions. β-galactosidase activities were used to normalize the reported luciferase activities. All experiments were independently replicated thrice to verify the results.

### Statistical analysis

All in vitro experiments were performed in triplicate, and differences between groups were analyzed using one-way ANOVA followed by Duncan’s multiple range test using IBM SPSS software (SPSS 25.0 for Windows; IBM, Chicago, IL, USA). Data for each group (*N *= 3) were expressed as mean ± standard error of mean (SEM). Statistical significance was set at *p *< 0.05.

## Results

### Analysis of gga-miR-200a-3p structure and candidate gene predictions

**Figure 1:**
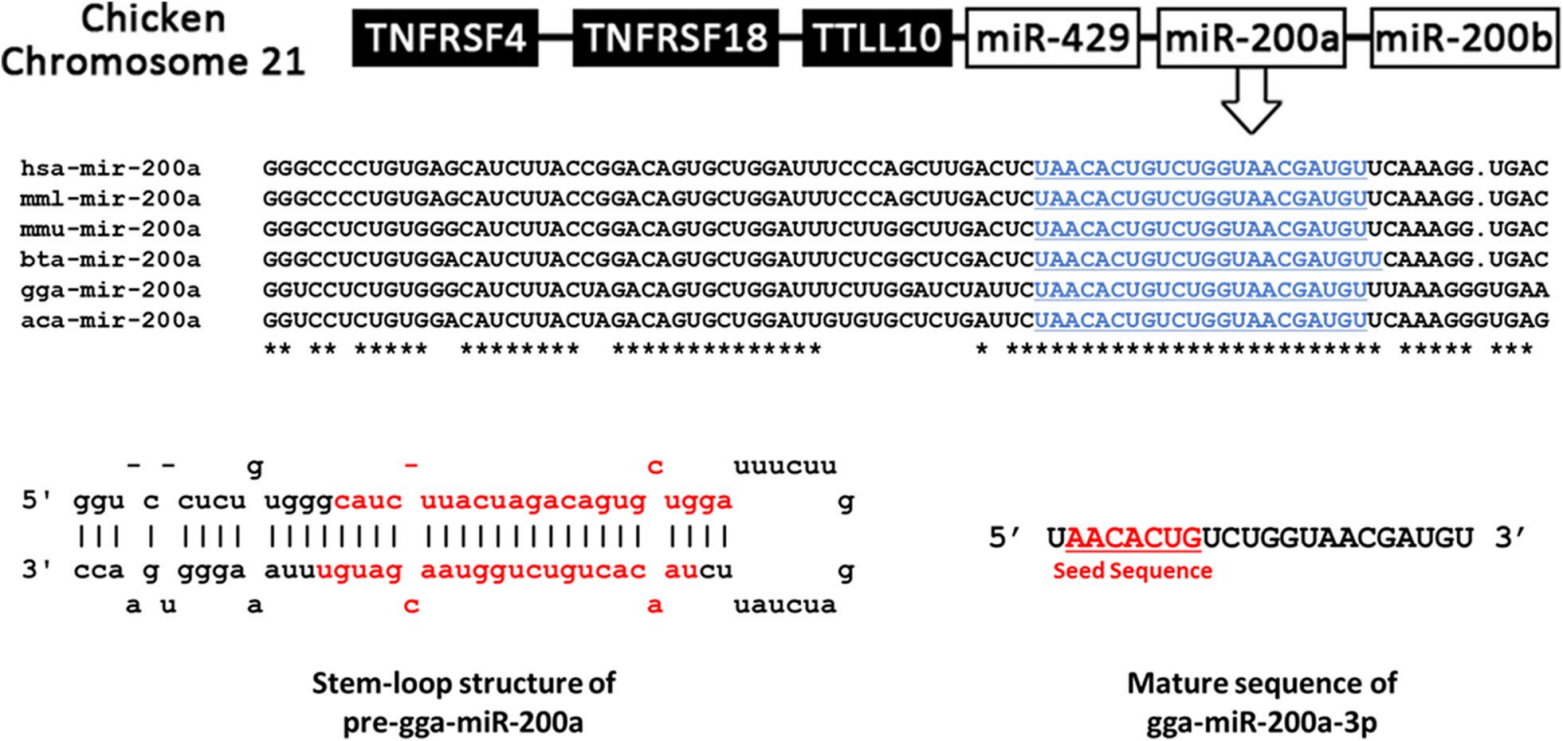
**Chromosomal position of chicken miR-200a and comparison of miR-200a sequences among several species.** Chicken miR-200a is located between miR-429 and miR-200b on chromosome 21. MiR-200a-3p is highly conserved across many species (hsa, *Homo sapiens*; mml, *Macaca mulatta*; mmu, *Mus musculus*; bta, *Bos taurus*; gga, *Gallus gallus*; Aca, *Anolis carolinensis*). The mature miR-200a-3p sequences of each species are in underlined in blue font and seed sequences are in underlined red font.

**Figure 2:**
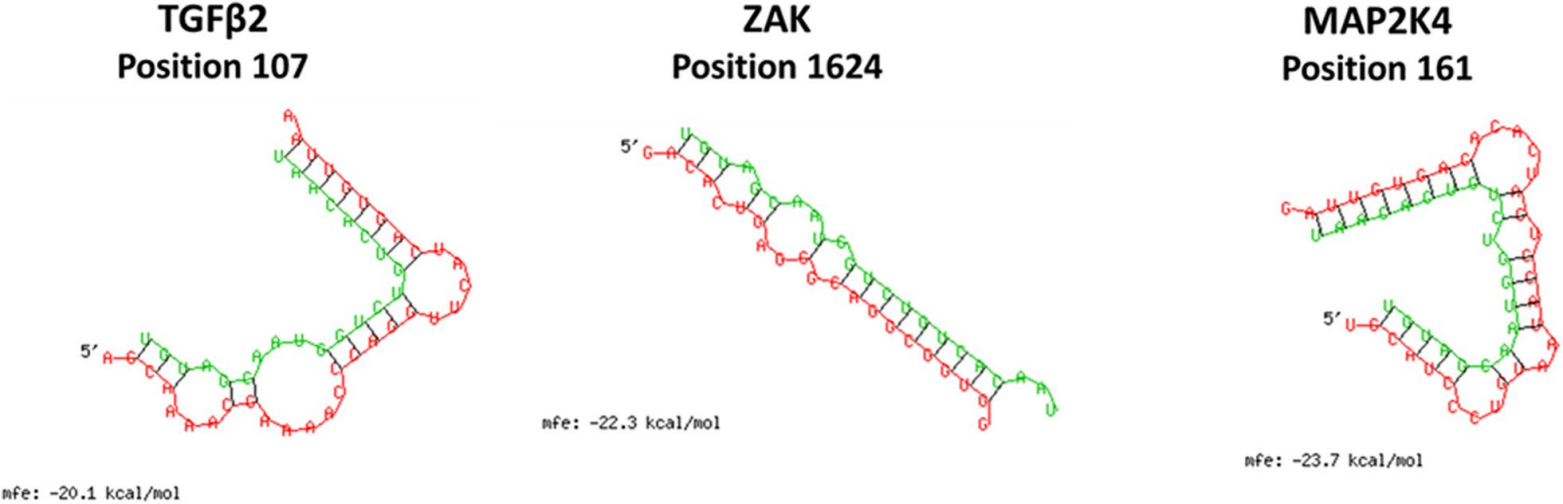
**Predicted binding structure and minimum free energy (mfe) between gga-miR-200a-3p and 3′-UTR of candidate target genes using RNA-hybrid algorithms available online.** Green lines and letters represent mature miRNA sequences and red lines and letters represent partial 3′-UTR sequences of target genes. “Position” means that the binding start sites from the start of 3′-UTR.

Gga-miR-200a-3p seed sequences among 6 species, human, monkey, mouse, cow, chicken and lizard were completely identical, indicating that these were highly conserved across vertebrates. In chicken, *gga*-*miR*-*200a*-*3p* was located between *gga*-*miR*-*429* and *gga*-*miR*-*200b*, on chromosome 21 (Figure 1). The miRDB algorithm, which was used for miRNA target gene prediction, revealed a total of 555 candidate target genes of gga-miR-200a-3p, primarily based on the sequence of gga-miR-200a-3p. Next, the genes were sorted based on target score, whereby 102 candidate target genes were found to display scores of over 90 (Additional file 2). Among them, four genes were involved in immune-related pathways, as predicted by DAVID and KEGG tools, among them, three were involved in the MAPK signaling pathway. These three genes, sterile alpha motif and leucine zipper containing kinase (*ZAK*); transforming growth factor beta 2 (*TGFβ2*); and mitogen-activated protein kinase kinase 4 (*MAP2K4*), were selected for further experimental validation. The binding sites between 3′-UTR of each candidate and gga-miR-200a-3p were predicted by the RNA-hybrid software tool, based on seed matches between the two molecules (nucleotides 2–8 of the miRNA and 3′-UTR of candidate genes) (Figure 2).

### Gga-miR-200a-3p and the expression of its target genes in two highly inbred chicken lines

**Figure 3:**
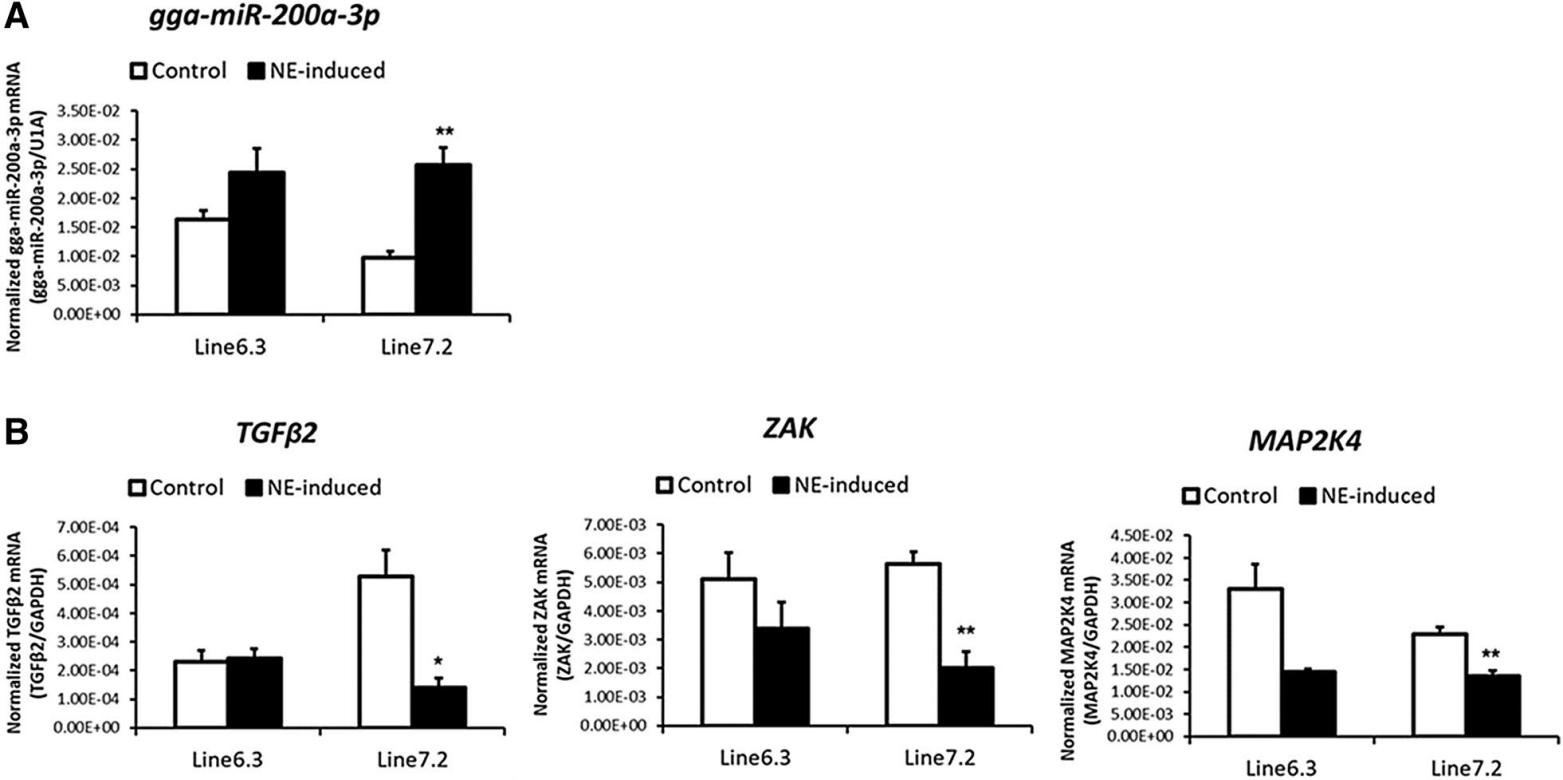
**The expression profile of gga-miR-200a-3p a and its candidate target genes b in IML derived from line 6.3 and line 7.2.** The expression levels of genes and miRNA were normalized to that of the glyceraldehyde-3-phosphate dehydrogenase (GAPDH) and U1A, respectively. Significant differences in gene expression levels between control and treatment (NE-induced) were analyzed by the Student’s *t* test and indicated as follows: * = *P* < 0.05 and ** = *P* < 0.01. Error bars indicate the SEM of technical replicates that were done in triplicate.

To determine the expression of gga-miR-200a-3p and putative target genes associated with NE-induction, qRT-PCR was carried using cDNA of IML samples from NE-afflicted and control groups (both MD-resistant line 6.3 and MD-susceptible line 7.2). On day 6 following NE-induction, the expression of gga-miR-200a-3p in IML was significantly increased in line 7.2, but not in line 6.3 (Figure 3A). Furthermore, the expression of all three candidate target genes was significantly downregulated only in line 7.2 (Figure 3B). These results indicate that expression of each candidate target gene was negatively correlated with that of gga-miR-200a-3p in line 7.2.

### Changes in target gene expression upon treatment with synthetic chicken miR-200a and LPS in chicken macrophage cell line

**Figure 4:**
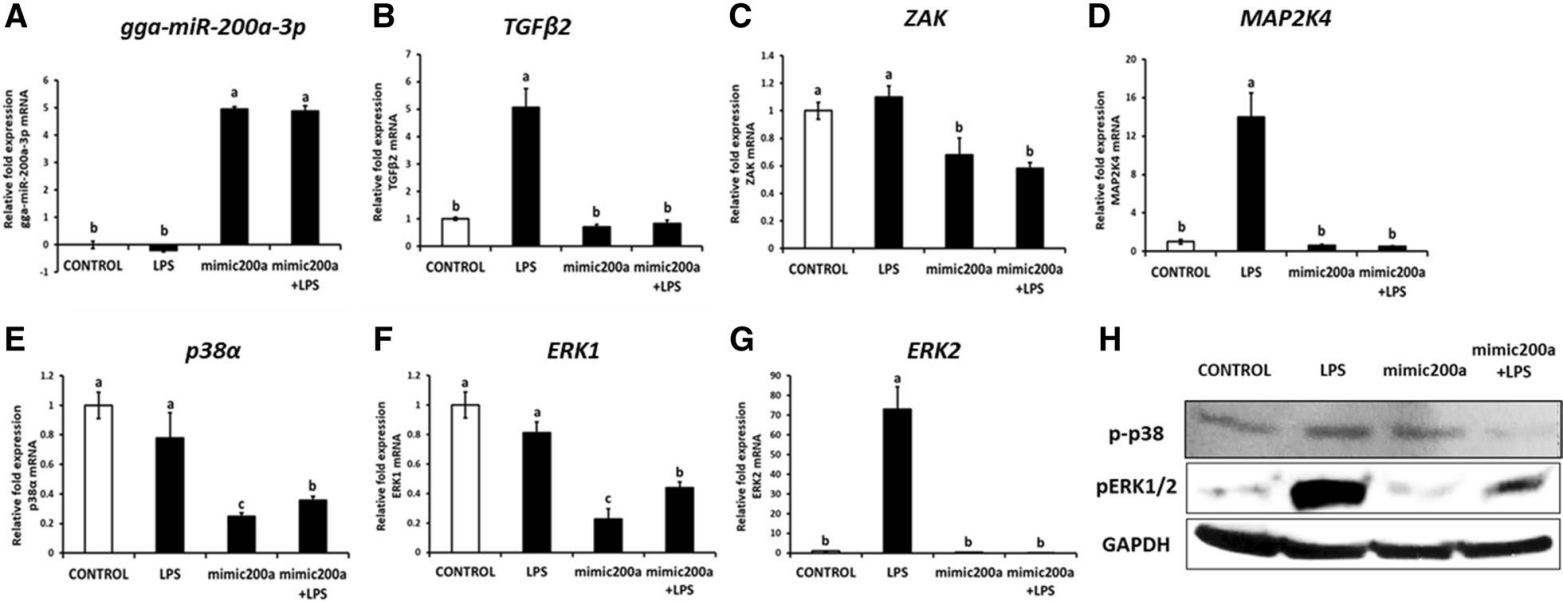
**Gga-miR-200a-3p repressed the expression of the immune target genes related to MAPK signaling pathway.** The expression profile of gga-miR-200a-3p **A** its candidate target genes. **B**–**D** The signaling molecules of MAPK signaling pathway **E**–**G** and Western blot analysis of the signaling molecules **H** in the HD11 chicken macrophage cell line treated with mimic miRNA and LPS.Expression data were transformed to a relative fold expression value in arbitrary units (AU) using the control expression level as a calibrator. Results are expressed as the mean ± SEM (*n* = 3) of three independent experiment.

In order to investigate whether the expression of candidate target genes can be altered by overexpressing gga-miR-200a-3p upon LPS stimulation—an endotoxins stimulus, we transfected gga-miR-200a-3p mimic into the HD11, and treated the cells with LPS from *S.E*. The expression of gga-miR-200a-3p in mimic miRNA transfected cells alone and in cells treated with LPS following mimic miRNA transfection, significantly increased compared to that of the control, indicating that gga-miR-200a-3p was successfully induced (Figure 4A). Among the three candidate target genes, the expression of *ZAK* mRNA decreased significantly in cells transfected with mimic miRNA alone (Figure 4C), while the expression of *TGFβ2, ZAK* and *MAP2K4* mRNA in cells treated with LPS following mimic miRNA transfection was strongly decreased (*p *<  0.05) relative to that in cells stimulated using only LPS (Figures 4B–D). However, the expression of gga-miR-200a-3p and its target gene *ZAK* displayed no changes in response to LPS from *S.E* compared to the control (Figures 4A, C), whereas significant increases were observed in the expression of *TGFβ2* and *MAP2K4* (fivefold and 14-fold, respectively) upon LPS treatment (Figures 4B, D). These results indicate that overexpression of gga-miR-200a-3p may down-regulate the expression of *TGFβ2*, *ZAK*, and *MAP2K4*.

### In vitro target gene validation of chicken miR-200a-3p in HD11 chicken macrophage cell line

**Figure 5:**
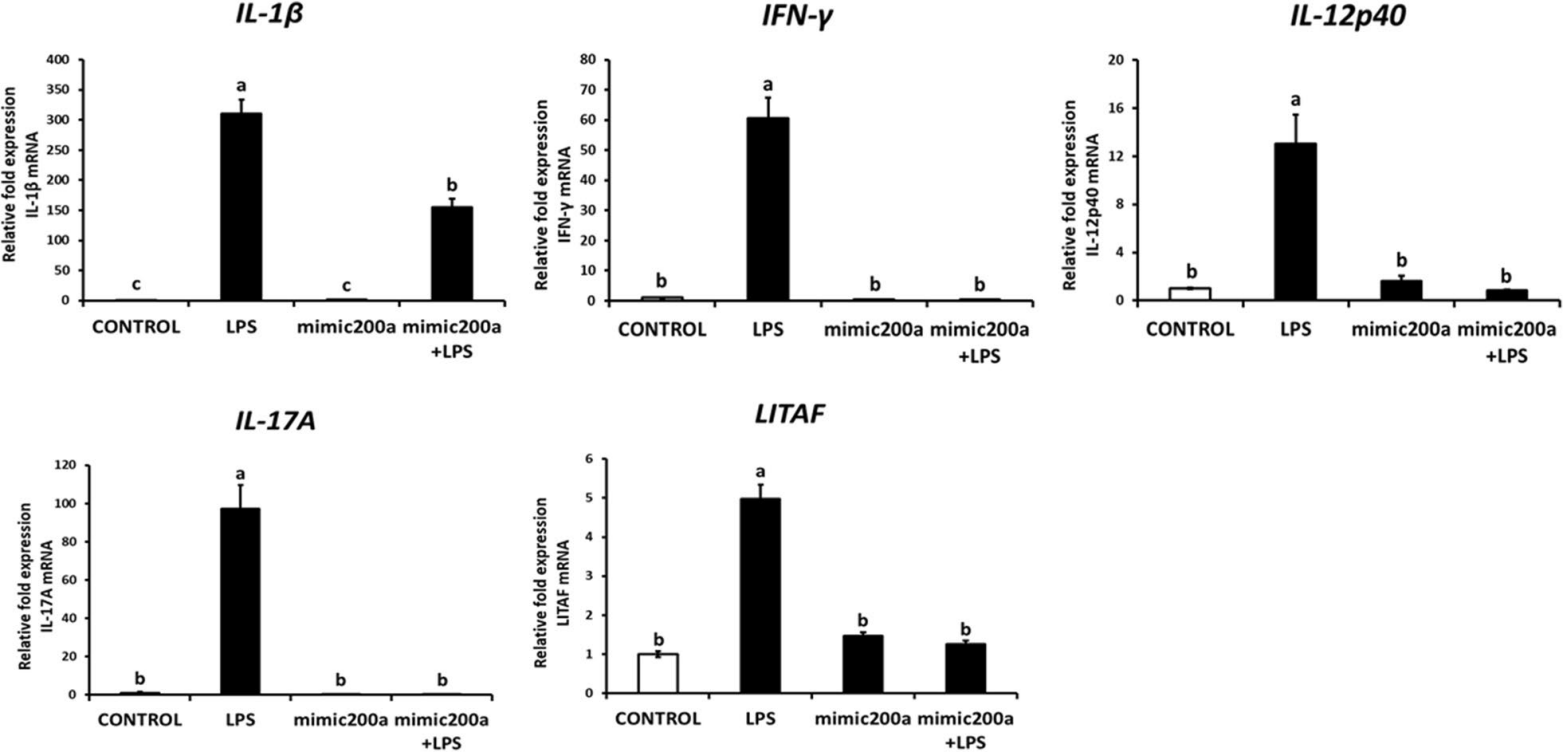
**Expression profile of cytokine genes involved in the MAPK signaling pathway of the HD11 chicken macrophage cell line treated with mimic miRNA and LPS.** Data are expressed as each mRNA level normalized against the GAPDH mRNA level. Data are expressed as the mean ± SEM (*n* = 3) of three independent experiments.

To identify whether miR-200a represses candidate target genes by targeting their predicted seed region in 3′-UTR site, a luciferase reporter assay and a dual fluorescence reporter assay were performed in HD11 cells.Each target gene site complementary to gga-miR-200a-3p was predicted using the RNA-hybrid computational analysis program (Figure 2). “TGFβ2/EGFP” eGFP reporter vectors were constructed (Figure 5A). Following transfection of miR200a/DsRed and/or TGFβ2/EGFP vector, eGFP and DsRed protein expression were analyzed by fluorescence microscopy. In cells co-transfected with both miR200a/DsRed and TGFβ2/EGFP constructs, DsRed and GFP fluorescence was significantly reduced compared to that in the control (Figure 5B). These results indicate that gga-miR-200a-3p might repress *TGFβ2* by targeting its 3′-UTR.

Structures of the luciferase reporter vectors including *TGFβ2*, *MAP2K4*, and *ZAK* were constructed. In addition, the DsRed reporter vector constructed as named miR200a/DsRed (Figure 5A). The miR200a/DsRed construct was co-transfected into the HD11 cell line along with a “Gene”/Luc vector or an empty luciferase vector as a control. Target gene expression patterns indicated that luciferase activity was significantly decreased when miR200a/DsRed was co-transfected with TGFβ2/Luc, ZAK/Luc or MAP2K4/Luc, compared with their control groups (Figure 5C). These results suggest that gga-miR-200a-3p may directly target *TGFβ2*, *MAP2K4*, and *ZAK*.

### Gga-miR-200a-3p inhibits the MAPK signaling pathway

**Figure 6:**
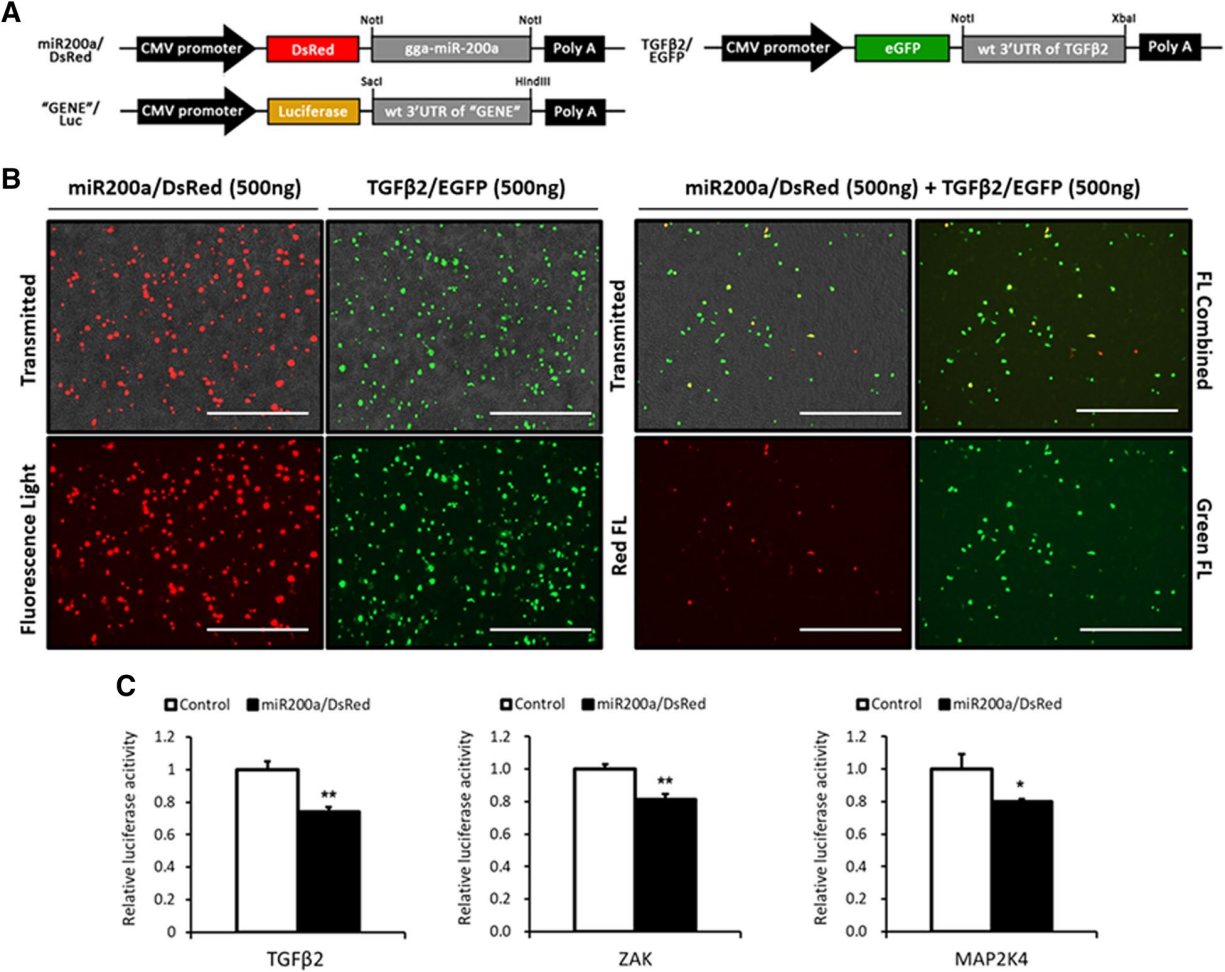
**Constructed vectors for this study and in vitro target validation of gga-miR-200a-3p. A** Schematic diagrams of expression vector for DsRed with gga-miR-200a-3p, eGFP with partial 3′-UTR of *TGFβ2* and Luciferase with partial 3′-UTR of selected genes. The miR200a/DsRed vector expresses both red fluorescence protein (RFP) and gga-miR-200a-3p; TGFβ2/EGFP vector expresses both enhanced green fluorescence protein (eGFP) and cloned 3′-UTR of *TGFβ2*. The “Gene”/Luc vectors express both luciferase and cloned 3′-UTR of *TGFβ2*, *MAP2K4*, or *ZAK* and named as TGFβ2/Luc, MAP2K4/Luc, and ZAK/Luc, respectively. The two restriction sites for cloning are indicated at both sides of inserted gene. **B** miR-200a/DsRed and TGFβ2/eGFP were co-transfected into HD11 chicken macrophage cell line in a dual fluorescence reporter. After 48 h of co-transfection, eGFP and DsRed expression were examined under a fluorescence microscope. White bars indicate 400 µm in length. **C** Luciferase reporter assay conducted for 24 h following co-transfection of “Gene”/Luc vectors with miR200a/DsRed or luciferase vector with miR200a/DsRed as the control in the HD11 chicken macrophage cell line. Results (mean ± SEM) are representative of three independent experiments.

Extracellular signal-regulated protein kinase (ERK) and the p38 MAP kinase are the two major MAP kinases that significantly contribute to innate immune response [31]. The levels of *p38α* and *ERK1* mRNA in mimic-miR-200a transfected groups were down-regulated by fivefold compared to that in the control. While no differences were observed between mimic-miR-200a-transfected samples and the control with respect to *ERK2* expression (Figures 4E–H). However, upon LPS treatment following transfection with mimic-miR-200a, the mRNA levels of *ERK1, p38α* were downregulated by twofold, and *ERK2* by 70-fold (Figures 4E–G). Further, we also analyzed the protein levels of p38 and ERK1/2 via Western blotting; the results showed that mimic-miR-200a inhibited the expression of p-p38 and pERK1/2 in the background of LPS treatment (Figure 4H). The downstream components of the MAPK signaling pathway, pro-inflammatory cytokines, including *IL*-*1β*, *IFN*-*γ,* and *IL*-*12p40* which are Th1 type cytokines; *IL*-*17A* which is a member of the Th17 cytokine family and the *LITAF*—proinflammatory mediator were significantly downregulated in the mimic-miR-200a transfected LPS-stimulated group compared with that in the only LPS treated group (Figure 6). Especially, the mRNA expression of *IFN*-*γ* and *IL*-*17A* which was remarkably repressed upon mimic-miR-200a overexpression, was strongly upregulated by approximately 60-fold and 100-fold, respectively, upon LPS treatment (Figure 6). These results indicate that mimic-200a may negatively regulate the MAP kinase-associated pathway and suppress the expression of proinflammatory cytokines in the HD11 chicken macrophage cell line.

## Discussion

An increasing number of studies are being conducted on the crucial role played by microRNAs, such as gga-miR-1306-5p [32], gga-miR-10a [33], gga-miR-130b-3p [34], in mediating the innate host response of chickens against pathogens. The current study investigated the biological function of gga-miR-200a-3p in the immune response. Analysis of *C.P*- and *E.M*- induced NE disease infections showed that miR-200a expression increased in chicken line 7.2, which was susceptible to ALV and MDV. By contrast, the expression of genes predicted to be gga-miR-200a-3p targets, and which were involved in the MAPK pathway showed an inverse correlation to that of miR-200a in chicken line 7.2, which is also susceptible to *C.P* and *E.M* pathogens.

In vitro experiments were conducted using the chicken macrophage cell line to validate the target genes of miR-200a. Similar to those from mammals, macrophages from avian species also play important roles in adaptive immunity by producing regulatory molecules such as cytokines, enzymes, and receptors. Avian macrophages also function in innate immunity by performing phagocytic and microbicidal functions [35].

Several target genes of miR-200a have been validated; these genes are associated with the immune signaling pathway including E-cadherin repressors, the wnt/β-catenin signaling pathway in gastric adenocarcinoma, *ZEB1/ZEB2* [36] and suppression of castration-resistant prostate cancer by inhibiting the activation of BRD4-mediated AR signaling in humans [37]. However, to the best of our knowledge, no target gene of miR-200a has been validated in chickens until now and no studies have been conducted on pathways mediated by miR-200a. The qRT-PCR data revealed that the overexpression of miR-200a repressed the expression of *MAP2K4*, *ZAK*, and *TGFβ2* mRNA. In addition, a luciferase reporter assay confirmed that chicken *MAP2K4*, *ZAK*, and *TGFβ2* were targeted by gga-miR-200a-3p. The results of the co-transfection experiments using constructs encoding eGFP-3′-UTR of *TGFβ2* and DsRed-miR-200a revealed that miR-200a directly bound the 3′-UTR of *TGFβ2* and silenced its expression. Our identification of chicken *TGFβ2* as a target of gga-miR-200a-3p is consistent with earlier reports in mice and humans [38, 39] and, to our knowledge, for the first time, two other genes, *MAP2K4* and *ZAK,* were also identified as the targets of gga-miR-200a-3p in this study. *ZAK* is a MAPK-kinase kinase (MKKK) which was involved in the activation of the ERK, p38 signaling pathways [40, 41]. In this study, LPS did not induce the expression of ZAK at the transcriptional level, but it did induce *MAP2K4* at the transcriptional level p-p38 and pERK1/2 at the translational level in the chicken macrophage cell line. Importantly, *MAP2K4* has been demonstrated to be a direct activator of MAP kinases that promote human prostate cancer metastasis [42] and act as prognostic markers of osteosarcoma tumorigenesis [43]. Although LPS did not successfully induce mRNAs level of *p38α* and *ERK1* in MAPK signaling pathway, the target genes of gga-miR-200a-3p might perform a vital function as inducers in the MAPK signaling pathway, while gga-miR-200a-3p was identified as its inhibitor.

The MAPK pathway is activated by many kinds of stimuli including endotoxic lipopolysaccharides (LPS), hyperosmolarity [44], proinflammatory cytokines and factors such as IL-1β, and PAF [45, 46] and viral infections [47]. In the present study, activation of the MAPK signaling pathway was induced by LPS stimulation. Additionally, gga-miR-200a-3p also inhibited the activity of the MAPK signaling pathway by suppressing MAPK signaling members of families, including *p38* and *ERK1/2,* to mediate the production of proinflammatory cytokines. Proinflammatory cytokines, *IL*-*1β*, *IL*-*12p40*, and *IFN*-*γ*, were induced by Th1 type cells; Th17 type cells induce following molecules such as *IL*-*17A* and *LITAF* which function as an inflammatory mediator [48] that were all up-regulated upon LPS treatment in our study. These proinflammatory cytokines were triggered due to host immune response via activation of the MAPK signaling pathway [49] which was shown to be repressed by gga-miR-200a-3p.

In conclusion, we demonstrated that gga-miR-200a-3p, a type of endogenous small noncoding RNA, is involved in transcriptional and translational regulation of genes related to the MAPK signaling pathway in chicken. The result indicates that susceptible line 7.2 to NE disease might be altered by the upregulation of gga-miR-200a-3p which suppressed the target genes, *MAP2K4*, *ZAK* and *TGFβ2*, which are involved in the MAPK signaling pathway, leading to a cascade inhibition of downstream kinase signaling molecules such as *ERK1/2*, *p38* and proinflammatory cytokines in chicken macrophage cell line. These results provide an understanding of the biological functioning of gga-miR-200a-3p in the host innate immune response and mechanisms underlying the gga-miR-200a-3p-mediated MAPK signaling pathway upon NE induction in chicken.

## Supplementary information

**Additional file 1:** **Primer sequences used for quantitative real-time PCR and cloning.**

**Additional file 2:** **List of putative target genes of gga-200a-3p predicted by miRDB.** Genes involved in immune-related pathway were identified using DAVID functional annotation tool, and KEGG pathway.

## Data Availability

All data generated or analyzed during this study are included in this published article and its supplementary information files.

## Abbreviations

MAPK signaling pathway: mitogen-activated protein kinase signaling pathway
TGFβ2: transforming growth factor beta-2
MAP2K4: mitogen-activated protein kinase kinase 4
ZAK: the sterile alpha motif and leucine zipper containing kinase
ERK1/2: extracellular signal-regulated protein kinases 1 and 2
LPS: lipopolysaccharide
IL: interleukin
LITAF: lipopolysaccharide-induced tumor necrosis factor-alpha factor
