# Supplementary material for: MicroRNA gga-miR-200a-3p modulates immune response via MAPK signaling pathway in chicken afflicted with necrotic enteritis
Source: Vet Res. 2020 Feb 3;51:8. doi: 10.1186/s13567-020-0736-x (PMC6998359; doi:10.1186/s13567-020-0736-x)
Supplement: Supplementary file 1 — Additional file 1: Primer sequences used for quantitative real-time PCR and cloning. [file 13567_2020_736_MOESM1_ESM.docx]

# **Additional file 1.** Primer sequences used for quantitative real-time PCR and cloning.

| **Genes** | **Primer sequence (5'-3')  Forward (F) and Reverse (R) primers** | | **Product length (bp)** | **Assay** | **Reference** |
| --- | --- | --- | --- | --- | --- |
| *gga-mir-200a-3p | F | TAACACTGTCTGGTAACGATGT | - | Real-time PCR | MIMAT0001171 |
|  | R | Universal Primer |  |  |  |
| U1A | F | CTGCATAATTTGTGGTAGTGG | - | Real-time PCR | V00444 |
|  | R | Universal Primer |  |  |  |
| ZAK | F | GGAAGCTCCAGTCAAAGTAA | 150 | Real-time PCR | XM_421996 |
|  | R | ATCCATGGAAAAGTACCCAC |  |  |  |
| TGFβ2 | F | AAGCTACCTGACTCCATTTG | 141 | Real-time PCR | NM_001031045 |
|  | R | GCATTGCGATTCAAGTGTTA |  |  |  |
| MAP2K4 | F | CCAAAAATATGTCGCGTTGA | 117 | Real-time PCR | XM_004946065 |
|  | R | TACAGGACGCCTAGTTAAGA |  |  |  |
| GAPDH | F | TGCTGCCCAGAACATCATCC | 142 | Real-time PCR | NM_204305 |
|  | R | ACGGCAGGTCAGGTCAACAA |  |  |  |
| gga-mir-200a | F | GCGGCCGCCACTTTCATTAGTTTTCACGGA | 354 | Luciferase Reporter Assay | MIMAT0001171 |
|  | R | GCGGCCGCGCTTCTTTGTCACGAGTATTTA |  |  |  |
| TGFβ2 | F | CGAGCTCGGATACCCCAGAAACAGCGT | 430 | Luciferase Reporter Assay | NM_001031045 |
|  | R | CCAAGCTTGTAAGGCACGGGACAGATAC |  |  |  |
| MAP2K4 | F | GAGCTCTATTGCTGCTACGTCAAACT | 562 | Luciferase Reporter Assay | XM_004946065 |
|  | R | AAGCTTGCAACAGAATTTCATAGCGG |  |  |  |
| ZAK | F | GAGCTCTAAAGGAAGCTGAACCCAAA | 832 | Luciferase Reporter Assay | XM_421996 |
|  | R | AAGCTTAGATCAGTTGCTCTTGCTTT |  |  |  |
| gga-mir-200a | F | GCGGCCGCCACTTTCATTAGTTTTCACGGA | 354 | Dual Fluorescence assay | MIMAT0001171 |
|  | R | GCGGCCGCGCTTCTTTGTCACGAGTATTTA |  |  |  |
| TGFβ2 | F | GCGGCCGCTACCCCAGAAACAGCGTGA | 630 | Dual Fluorescence assay | NM_001031045 |
|  | R | GCTCTAGACTTGCGTTCCTTTAGGCTTT |  |  |  |
| p38α | F | CTGCACGTGGTCATCTGTAAGT | 116 | Real-time PCR | XM_419263 |
|  | R | TCACCCCTGCCAAGTCTCT |  |  |  |
| ERK1 | F | GCAAGCTTTAGCCCATCCA | 97 | Real-time PCR | NM_204150 |
|  | R | GTCATCCAATTCCATATCAAACTT |  |  |  |
| ERK2 | F | CATCGCGACCTCAAACCTTC | 92 | Real-time PCR | AAK56503 |
|  | R | TCCGGATCTGCAACACGAG |  |  |  |
| IL-1β | F | TGGGCATCAAGGGCTACA | 244 | Real-time PCR | NM_204524 |
|  | R | TCGGGTTGGTTGGTGATG |  |  |  |
| IFN-γ | F | TGAAGAGTTCATTCGCGGCT | 106 | Real-time PCR | NM_205149.1 |
|  | R | AACAACCTTCCTGATGGCGT |  |  |  |
| IL-17A | F | TGTCTCCGATCCCTTGTTCT | 104 | Real-time PCR | AM773756 |
|  | R | GTCCTGGCCGTATCACCTT |  |  |  |
| LITAF | F | TGTGTATGTGCAGCAACCCGTAGT | 229 | Real-time PCR | AY765397 |
|  | R | GGCATTGCAATTTGGACAGAAGT |  |  |  |
| IL-12p40 | F | AGATGCTGGCAACTACACCTG | 123 | Real-time PCR | NM_213571 |
|  | R | CATTTGCCCATTGGAGTCTAC |  |  |  |

* Forward primer of miRNAs is designed to identical to the entire mature miRNA sequence. The underlined sequences represent the *Not* I, *Xba* I, *Sac* I and *Hind* III restriction enzyme loci used for cloning; GCGGCCGC for *Not* I, TCTAGA for *Xba* I, GAGCTC for *Sac* I and AAGCTT for *Hind* III.
