# Supplementary material for: MicroRNA gga-miR-200a-3p modulates immune response via MAPK signaling pathway in chicken afflicted with necrotic enteritis
Source: Vet Res. 2020 Feb 3;51:8. doi: 10.1186/s13567-020-0736-x (PMC6998359; doi:10.1186/s13567-020-0736-x)
Supplement: Supplementary file 2 — Additional file 2: List of putative target genes of gga-200a-3p predicted by miRDB. Genes involved in immune-related pathway were identified using DAVID functional annotation tool, and KEGG pathway. [file 13567_2020_736_MOESM2_ESM.docx]

# **Additional file 2**. List of putative target genes of gga-miR-200a-3p predicted by miRDB. Genes that involved in immune-related pathway was found from DAVID functional annotation tool, and KEGG pathway.

| **Target Score** | **Gene Symbol** | **Immune-related pathway** |
| --- | --- | --- |
| 100 | CCDC6 |  |
| 100 | ZEB1 |  |
| 100 | CAMSAP2 |  |
| 100 | ATP8A1 |  |
| 100 | CHST4 |  |
| 100 | ATP6V1B2 |  |
| 100 | FAM188A |  |
| 99 | OSBPL11 |  |
| 99 | REV3L |  |
| 99 | SOGA2 |  |
| 99 | ETNK1 |  |
| 99 | ELAVL2 |  |
| 99 | GLS |  |
| 99 | MOBKL2B |  |
| 99 | CAPN6 |  |
| 99 | PPM1L |  |
| 99 | ZEB2 |  |
| 99 | KIAA1549 |  |
| 99 | CTNND2 |  |
| 98 | TET1 |  |
| 98 | ATXN7 |  |
| 98 | GPRIN3 |  |
| 98 | BRD3 |  |
| 98 | SCN2A |  |
| 98 | DR1 |  |
| 98 | CYP26B1 |  |
| 97 | HMG20A |  |
| 97 | C13H5ORF24 |  |
| 97 | UNC119B |  |
| 97 | STAG1 |  |
| 97 | SNX8 |  |
| 97 | TSHZ3 |  |
| 97 | ZAK | MAPK signaling pathway |
| 96 | ANKRD44 |  |
| 96 | ULK2 |  |
| 96 | ARHGEF7 |  |
| 96 | TMEM170B |  |
| 96 | DDX46 |  |
| 95 | TTLL7 |  |
| 95 | GPAM |  |
| 95 | LOC100859766 |  |
| 95 | PTPRG |  |
| 95 | NECAP2 |  |
| 95 | DMBX1 |  |
| 95 | ABL2 |  |
| 95 | PITX2 | TGF-beta signaling pathway |
| 95 | HIPK3 |  |
| 95 | RNF38 |  |
| 94 | DCUN1D3 |  |
| 94 | CLASP2 |  |
| 94 | GOLT1B |  |
| 94 | SIPA1L2 |  |
| 94 | TMEM56 |  |
| 94 | C2H9ORF5 |  |
| 94 | CHP1 |  |
| 94 | WDFY3 |  |
| 94 | ITSN1 |  |
| 94 | TGFB2 | MAPK signaling pathway, TGF-beta signaling pathway |
| 94 | FAM8A1 |  |
| 94 | TSC1 |  |
| 94 | ARHGAP28 |  |
| 93 | NUDT12 |  |
| 93 | CDK17 |  |
| 93 | SEMA6A |  |
| 93 | VGLL3 |  |
| 93 | MYH10 |  |
| 93 | RNMT |  |
| 93 | TAF12 |  |
| 93 | DPY19L4 |  |
| 93 | IGSF5 |  |
| 93 | LOC770321 |  |
| 92 | ATRN |  |
| 92 | CDK13 |  |
| 92 | CLSPN |  |
| 92 | LRRC8B |  |
| 92 | HAS2 |  |
| 92 | ELMOD1 |  |
| 92 | SRGAP1 |  |
| 92 | DOCK4 |  |
| 92 | PCDH9 |  |
| 91 | GPR137C |  |
| 91 | FAM91A1 |  |
| 91 | C8ORF42 |  |
| 91 | THSD7A |  |
| 91 | CNTN1 |  |
| 91 | ASXL1 |  |
| 91 | KAT6B |  |
| 91 | PDS5B |  |
| 91 | AK2 |  |
| 91 | TCF12 |  |
| 91 | C8H1ORF21 |  |
| 91 | FAM189A1 |  |
| 91 | UBXN2B |  |
| 91 | ZC3H12B |  |
| 91 | MAP2K4 | MAPK signaling pathway, Toll-like receptor signaling pathway |
| 91 | SCHIP1 |  |
| 90 | ZC3H12C |  |
| 90 | TMTC4 |  |
| 90 | ZCCHC24 |  |
| 90 | AFF4 |  |
| 90 | BICD2 |  |
| 90 | FAM168B |  |
